# Chronically Elevated *O*-GlcNAcylation Limits Nitric Oxide Production and Deregulates Specific Pro-Inflammatory Cytokines

**DOI:** 10.3389/fimmu.2022.802336

**Published:** 2022-04-01

**Authors:** Lara K. Abramowitz, John A. Hanover

**Affiliations:** Laboratory of Cell and Molecular Biology, National Institute for Diabetes and Digestive and Kidney Diseases, National Institutes of Health, Bethesda, MD, United States

**Keywords:** *O*-GlcNAcylation, *O*-GlcNAcase, inflammation, iNOS, macrophages, cytokines

## Abstract

Inflammation is the immune response to harmful stimuli, including pathogens, damaged cells and toxic compounds. However, uncontrolled inflammation can be detrimental and contribute to numerous chronic inflammatory diseases, such as insulin resistance. At the forefront of this response are macrophages, which sense the local microenvironment to respond with a pro-inflammatory, M1-polarized phenotype, or anti-inflammatory, M2-polarized phenotype. M1 macrophages upregulate factors like pro-inflammatory cytokines, to promote inflammatory signaling, and inducible Nitric Oxide Synthase (iNOS), to produce nitric oxide (NO). The generated NO can kill microorganisms to protect the body, but also signal back to the macrophage to limit pro-inflammatory cytokine production to maintain macrophage homeostasis. Thus, the tight regulation of iNOS in macrophages is critical for the immune system. Here, we investigated how elevation of the nutrient-sensitive posttranslational modification, *O*-GlcNAc, impacts M1 polarized macrophages. We identified increased gene expression of specific pro-inflammatory cytokines (*Il-6, Il-1β, Il-12*) when *O*-GlcNAc cycling was blocked. We further uncovered an interaction between *O-*GlcNAc and iNOS, with iNOS being an OGT target *in vitro*. Analysis of M1 polarized bone marrow derived macrophages deficient in the enzyme that removes *O*-GlcNAc, *O*-GlcNAcase (OGA), revealed decreased iNOS activity as measured by a reduction in NO release. Further, elevated *O*-GlcNAc acted on *Il-6* expression through the iNOS pathway, as iNOS inhibitior L-NIL raised wildtype *Il-6* expression similar to OGA deficient cells but had no further effect on the hyper-*O*-GlcNAcylated cells. Thus *O*-GlcNAc contributes to macrophage homeostasis through modulation of iNOS activity.

## Introduction

The immune system is able to defend against internal and external insults by promoting inflammatory processes. However, inflammation can also be potentially harmful to the body and needs to be tightly regulated to avoid tissue damage and long-term disease. Macrophages are a heterogenous population of immune cells that act as the main player in the first-line of defense against pathogens, modulating homeostatic and inflammatory responses. The wide range of macrophage functions relies on the ability to sense and respond to the microenvironment. Through the regulation of specific gene expression programs, in conjunction with metabolic adaptations, macrophages are able to modulate their functions and respond to stimuli.

Macrophages are generally described as classically activated, pro-inflammatory M1 macrophages or alternatively activated, anti-inflammatory M2 macrophages. Pro-inflammatory M1 macrophages are induced by microbial products, like lipopolysaccharide (LPS) and other toll like receptors (TLRs) ligands, or by cytokines secreted by T_H_-1 lymphocytes, like interferon gamma (IFN-γ). M1 macrophages are characterized by their ability to kill pathogens and present antigens to T-lymphocytes. Thus, they produce high levels of pro-inflammatory cytokines, like interleukin (IL)-6, IL-1β, IL-12 and tumor necrosis factor alpha (TNF-α) (1). Despite playing a critical role in the host defense against pathogen infection, tight regulation of these pro-inflammatory cytokines is critical to maintain proper homeostasis as deregulation has been associated with a number of autoimmune and inflammatory diseases. Therefore, understanding the underlying regulatory mechanisms of M1 pro-inflammatory cytokine production is of great importance.

Integral to M1 function is the expression and regulation of inducible Nitric Oxide Synthase (iNOS) which produces nitric oxide (NO) from L-arginine to kill invading pathogens. Unlike the other Nitric Oxide Synthases, neuronal Nitric Oxide Synthase (nNOS) and endothelial Nitric Oxide Synthase (eNOS), iNOS is not constitutively active and is only expressed when cells are stimulated with factors such as LPS or IFN-γ (2). Because of its critical role in the inflammatory response, iNOS expression and activity is strictly controlled. Transcriptionally, iNOS is regulated by the transcription factors NF-κB and STAT-1a (3). At the protein level, iNOS is regulated by tyrosine phosphorylation (4), substrate (L-arginine) availability, protein-protein interactions and auto-inactivation (2). Interestingly, iNOS is not only a key component of the inflammatory response and a hallmark of M1 macrophages, but NO also feeds back to limit expression of pro-inflammatory cytokines (5, 6). In fact, stimulated macrophages from iNOS deficient mice, or treatment of wildtype macrophages with an iNOS selective inhibitor, L-NIL [L-N^6^-(1-Iminoethyl)lysine dihydrochloride], enhanced expression of pro-inflammatory cytokines like IL-12 and IL-6 (5). Thus, iNOS is a key factor in balancing macrophage homeostasis.

Upon stimulation, inflammatory macrophages are characterized by their glycolytic metabolism. A number of studies have found that glucose availability impacts M1 polarization (7, 8), regulation of key inflammatory modulators like iNOS (9), and expression of inflammatory cytokines (10, 11). How nutrients specifically impact the inflammatory response remains an unanswered and important question in the field, as diseases with altered nutrient signaling, like diabetes, are well known to have an inflammatory component. For this reason, understanding how changes in the nutrient-sensing posttranslational modification, *O*-GlcNAcylation, impacts the inflammatory response of macrophages is of great interest with clinical significance. This single monosaccharide is attached to serines and threonines of intracellular proteins by the *O*-GlcNAc Transferase (OGT) enzyme, which uses the end-product of the hexosamine biosynthetic pathway (HBP), UDP-GlcNAc, as the sugar donor for protein modification. The HBP incorporates metabolites like glucose, amino acids, fatty acids, and nucleotides to form UDP-GlcNAc, thus levels of UDP-GlcNAc reflect the nutrient status of the cell. *O*-GlcNAc is a dynamic posttranslational modification and is removed by *O*-GlcNAcase (OGA). Although there have been investigations into how *O*-GlcNAc might impact M1 polarization and the inflammatory response, these studies do not come to a clear conclusion, with some published studies declaring *O*-GlcNAcylation as pro-inflammatory (12–14) and others concluding it is anti-inflammatory (15–17). These studies have either used acute chemical OGT or OGA inhibition or mice that have deleted OGT (15, 16). What remains unknown is how chronic high levels of *O*-GlcNAc, which mimic the signaling state of nutrient over-abundance, such as hyperglycemia, impacts key inflammatory components of M1 macrophages.

To model chronically elevated *O-*GlcNAc, we analyzed M1 stimulated bone marrow derived macrophages (BMDMs) from mice in which *Oga* has been deleted within the hematopoietic lineage (*Oga^Vav-Cre^* (18),). OGA deficient macrophages had significantly elevated gene expression of key pro-inflammatory cytokines like *Il-6*. Focusing on iNOS, we found that NO release was diminished in *Oga* knockout (KO) cells. Further, elevated *O-*GlcNAc impacted *Il-6* expression through the iNOS pathway, as inhibition of iNOS in wildtype cells using the inhibitor L-NIL increased *Il-6* expression similar to OGA deficiency alone, but had no further effect on the *Oga* KO macrophages. WGA pull-down analysis and *in vitro* OGT assays provided evidence that iNOS could be an OGT target. Together, these data indicated that through interaction with iNOS, elevated *O*-GlcNAc dampens NO production and contributes to deregulation of specific pro-inflammatory cytokines. Thus, balanced *O*-GlcNAc cycling is a key component for maintaining macrophage homeostasis.

## Methods

### Mice

The *Oga^Vav-Cre^* line was maintained as previously described (Abramowitz 2019). Both males and females were used in analysis. The animals were maintained according to the animal protocol # K023-LCBB-19 approved by the NIDDK Animal Care and Use Committee, National Institutes of Health.

### Cell Culture

Bone marrow was extracted from the femur and longbone of 6-12 week old mice and plated at a concentration of 2x10^6^/ml and cultured in BMDM media containing IMDM, 10% FBS, 10ng/ml Macrophage-Colony Stimulating Factor (m-CSF) (Gemini), 1% penicillin/streptomycin, 1% glutamine. Media was changed to fresh BMDM media on day 3. After 7 days in culture, cells were changed to M1 media containing IMDM, 10%FBS, 120-180 ng/ml LPS (Sigma), 50 ng/ml IFN-γ (Biolegend), 1% penicillin/streptomycin, 1% glutamine. For M2 polarizations, cells were changed to M2 media containing IMDM, 10% FBS, 10ng/ml IL-4 (Biolegend), 1% penicillin/streptomycin, 1% glutamine. After 24 hours polarization cells were collected for analysis. In experiments with L-NIL (Cayman Chemical), 40 uM of L-NIL was added to M1 media.

### Cell Viability

Cells were mixed in a 1:1 ration with .4% trypan blue and percent viability measured using a Nexcelcom Cellometer.

### Western Blot

Protein was extracted with T-PER tissue protein extraction reagent (Termo-Fisher). Lysates were run on a 4–12% Bis-Tris gel and transferred to a nitrocellulose membrane. Membranes were blocked with Odyssey blocking buffer (Li-Cor) for 1 hour, incubated overnight at 4°C with primary antibodies: anti-actin (Abcam ab1801), anti-*O*-GlcNAc (RL2) (Termo Fisher MA1-072), OGT (Santa Cruz H300), or iNOS (Invitrogen PA3-030A or Abcam ab178945). Secondary antibodies were Odyssey IR dye-conjugated (LI-Cor). Membranes were imaged and band intensities were analyzed with Odyssey imaging equipment.

### qRT-PCR

RNA was extracted using the RNeasy (II) mini kit (Qiagen) following manufacturers protocol, followed by DNAseI treatment (Invitrogen). cDNA was synthesized using the qScript kit (Quantbio) following manufacturer’s protocol. Fast SYBR Green Master Mix (Applied Biosystems) was used for amplification using 5 ng of cDNA and the appropriate primer (**Supplemental Table 1**) on a QuantStudio 3 instrument (Applied Biosystems). Each reaction was performed in technical triplicate. Relative gene expression was normalized to the geometric mean of *Rplp0* and *Eef2* (**Supplemental Table 1**).

### ELISA

Media samples taken from M1 cultures were diluted 1:10 for IL-6, 1:50 for IL-12 and undiluted for IL-1β, and measured by mouse IL-6, IL-12/IL-23p40 non allele specific or IL-1b/IL-1F2 quantikine ELISA kits (R&D systems), respectively, following the manufacturer’s protocol. Absorbances were measured on a POLARSTAR omega microplate reader (BMG Labtech).

### WGA Precipitation

Cell lysates were obtained using TPER (Thermofisher). Lysates were pooled from 3 independent samples of each genotype to obtain enough material for analysis. 10 μl/sample of agarose bound wheat germ agglutinin (WGA) (Vectorlabs) was washed three times in PBS containing 0.2% NP40. 100 μg of sample was added to the agarose beads and incubated overnight at 4°C. The next day, the beads were washed four times in PBS containing 0.2% NP-40 for 20 minutes each. Loading dye was added to the beads, boiled for 10 minutes, and processed as described above for western blot analysis.

### *In Vitro* OGT Assay

4 μg of purified OGT was incubated with 5-10ul of BL21(DE3) lysates containing recombinant mouse iNOS (Caymen chemical) or BL21(DE3) lysates without iNOS expression (negative control for iNOS specificity) or purified recombinant human CSNK2A1 (CKII, Abcam ab85981, positive control with known OGT target), UDP-GlcNAz or no UDP-GlcNAz (negative control for background banding) and OGT assay buffer (19) to 50ul. After a 2 hour incubation at 37°C, the reactions were centrifuged through a 10K filter (Millipore) to remove excess nucleotide-sugar. A Staudinger ligation was then performed by adding biotin-phosphine and incubating for 1 hour at 40°C. Loading dye was added to the reactions and boiled for 10 minutes. Samples were then processed as described above for western blot with the biotin detected using a streptavidin-conjugated IRdye from LICOR.

### Griess Reagent

Nitrite concentration was measured in media collected from cells after 24 hour incubation in M1 media using the Griess reagent kit (Cell Signaling) following manufacturer’s protocol.

### Statistics

Graphpad prism was used for all statistics. An unpaired t-test with Welch’s correction was used to determine significance as indicated in figure legends. A two-way ANOVA was used to compare levels of *O*-GlcNAc in M0 versus M1 conditions. P values less than 0.05 were considered statistically significant.

## Results

### OGA Deficient Naïve and Stimulated BMDMs Exhibit Higher Levels of *O*-GlcNAc

**Figure 1:**
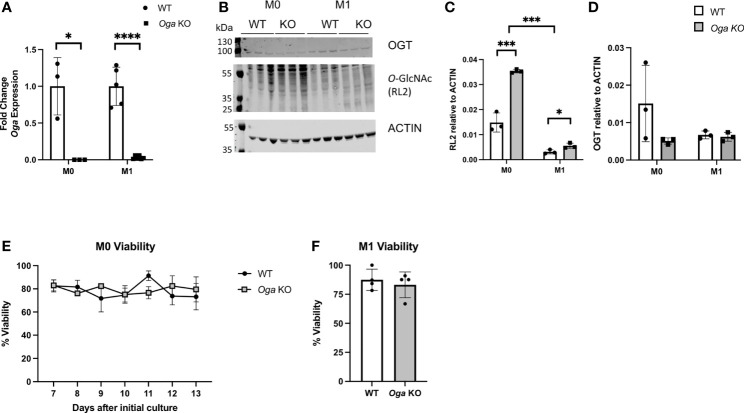
Increased *O*-GlcNAcylation in BMDMs derived from *Oga^Vav-Cre^* mice. BMDMs derived from *Oga^Vav-Cre^* (KO, grey bars) or wildtype (WT, white bars) mouse bone marrow were stimulated with LPS and IFN-γ (M1) for 24 hours or cultured in unsupplemented BMDM media (M0). **(A)** *Oga* gene expression was measured in M0 and M1 macrophages. Expression was normalized to the geometric mean of *Rplp0* and *Eef2* and represented as fold change, N = 3-5. **(B–D)** *O*-GlcNAc (using the RL2 antibody) and OGT levels were assessed in M0 and M1 macrophages, with a representative blot **(B)** and quantitation (C-D), N = 3. **(E, F)** Percent cell viability was measured using trypan blue starting 7 days after initial culture in BMDM media **(E)** and 1 day after M1 polarization **(F)**, N = 3-4. Error bars represent standard deviations. *p < 0.05, ***p < 0.005, ****p < 0.00005 as determined by student’s t-test, or 2-way ANOVA when comparing O-GlcNAc levels between M0 and M1.

In order to study the impact of chronic hyper-*O*-GlcNAcylation on macrophage function, we utilized mice in which *Oga* had been deleted in hematopoietic stem cells (HSC) (18). For these studies, bone marrow cells were extracted from *Oga^Vav-Cre^* mice along with their wildtype littermates and cultured in macrophage-colony stimulating factor (m-CSF) for 7 days to obtain a population of BMDMs. The BMDMs were then stimulated *in vitro* with LPS and IFN-γ to induce M1 polarization. To ensure loss of *Oga*, we assessed *Oga* transcription in BMDMs before and after polarization. We were unable to detect *Oga* transcripts in these cells confirming *Oga* deletion (**Figure 1A**). Next, we assessed levels of global *O*-GlcNAc and OGT before and after stimulation in the OGA deficient and wildtype BMDMs. As anticipated, deletion of *Oga* resulted in a global increase in *O*-GlcNAcylation as compared to wildtype counterparts (**Figures 1B** **–D**). Similar to previous reports (15, 16), we found a decrease in overall *O*-GlcNAc levels upon M1 polarization of wildtype BMDMs (**Figures 1B, C**). This is consistent with the observation that products of the HBP decrease after LPS stimulation (15). Interestingly, *O-*GlcNAcylation level of the OGA deficient M1 polarized BMDMs were increased as compared to wildtype M1 polarized BMDMs, but still remained lower than M0 BMDMs (**Figures 1B, C**). We also assessed OGT expression as cells often compensate for changes in OGA with a coordinate change in OGT. We did not detect significant decreases in OGT protein (**Figures 1B, D**) nor transcript levels (**Supplementary Figure 1**) in the *Oga* KO cells as compared to wildtype BMDMs.

To further characterize the OGA deficient BMDMs, we assessed whether loss of OGA impacted cell viability. Viability of the mutant versus wildtype BMDMs was assessed daily starting at 7 days in culture with m-CSF through 13 days in culture with m-CSF. Both OGA deficient and wildtype BMDMs had similar levels of cell viability (**Figure 1E**). Next, we assessed viability 1 day after M1 polarization and again found similar cell viability in the *Oga* KO BMDMs as wildtype BMDMs (**Figure 1F**). These data confirm that the OGA deficient cells represent a good model for investigating the impact of chronically elevated *O*-GlcNAc on macrophages.

### Increase in Gene Expression of Specific Pro-Inflammatory Cytokines in OGA Deficient M1 Polarized BMDMs

**Figure 2:**
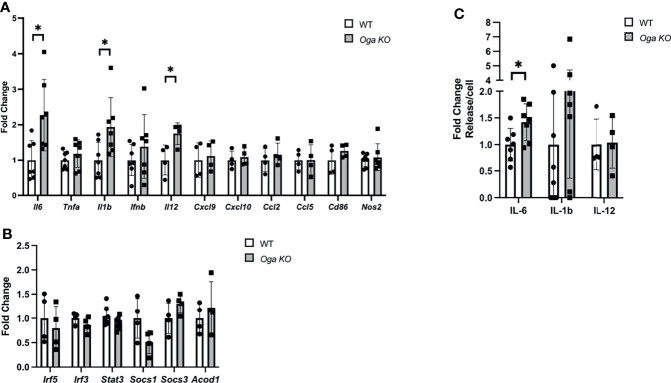
Increase in specific pro-inflammatory cytokine gene expression in M1 polarized OGA deficient BMDMs. **(A, B)** Gene expression of M1 markers **(A)** and transcription and metabolic regulators **(B)** in stimulated WT (white bars) and *Oga* KO BMDMs (grey bars), with respective genes indicated on the X-axis. Expression was normalized to the geometric mean of *Rplp0* and *Eef2* and represented as fold change. **(C)** ELISAs for IL-6, IL-1β and IL-12 were performed using media from M1 polarized WT and *Oga* KO BMDMs. Levels of respective ILs were normalized to cell counts and represented as fold change. N = 4-7. Error bars represent standard deviations. *p < 0.05 as determined by student’s t-test.

M1 macrophages are defined by a shift in transcriptional networks that promote proinflammatory cytokines, chemokines and metabolic regulators. Understanding the regulatory mechanisms of these factors is of great importance as uncontrolled inflammatory cytokine expression can lead to tissue damage and contribute to chronic disease. Previous reports indicated that nutrient availability could impact levels of inflammatory cytokine production (7–11). To assess the impact that *Oga* deletion had on the macrophage inflammatory response, we analyzed expression of key pro-inflammatory markers, including cytokines, chemokines, metabolic regulators and transcription factors. Interestingly, we found increased transcription of specific pro-inflammatory cytokines like *Il-6*, *Il-1β* and *Il-12* as compared to wildtype (**Figure 2A**). However, there was no change in other inflammatory markers like *Cxcl9*, *Ccl5*, *Cd86*, *Nos2* (encodes iNOS), etc. (**Figure 2A**), Further, we assessed gene expression levels of transcription factors known to regulate inflammation, and the metabolic regulator *Acod1* which functions to produce itaconate and mitochondrial reactive oxygen species. There were no detectable changes in these inflammatory regulators (**Figure 2B**). These data suggest that the impact of elevated *O-*GlcNAc on macrophages is downstream of more broad pro-inflammatory regulators.

To confirm that increased pro-inflammatory cytokine gene expression could have physiological consequences, we assessed levels of the deregulated cytokines in the media by ELISA. Similar to increased transcription of *Il-6*, we found increased levels of IL-6 secreted into the media (**Figure 2C**). However, no changes in secretion were detected for IL-1β and IL-12, although secretion levels of IL-1β were trending up for the *Oga* KO cells (**Figure 2C**). Thus, while deletion of *Oga* did not broadly increase all M1 markers, it did impact gene expression of specific pro-inflammatory cytokines and the secreted levels of IL-6.

After detecting increases in some pro-inflammatory markers, we next asked if increased *O*-GlcNAc would impact anti-inflammatory M2 macrophage polarization. For M2 polarization we treated the BMDMs with IL-4 for 24 hours and assessed transcription of M2 markers *Arg1*, *Il-10*, *Retn1a, chi313*, and *Cd206* (**Supplementary Figure 2**). No changes in expression of these markers were detected. This is consistent with previous reports in which deregulating *O*-GlcNAc through deletion of *Ogt* did not impact M2 polarization (15, 16).

### Hyper-*O*-GlcNAcylation Dampens Macrophage iNOS Activity

Because *Oga* deletion resulted in specific rather than broad impacts on the inflammatory response in macrophages we decided to focus on factors that might respond to *O*-GlcNAc signaling and act to fine-tune M1 homeostasis. Due to its sequence homology with eNOS, a known *O*-GlcNAc modified protein, and its ability to impact transcription of specific pro-inflammatory cytokines (5), we focused on iNOS. Interestingly, iNOS expression has been reported to be influenced by glucose and glucosamine (9), and transcription factors like NF-κB (20), STAT1 (21, 22) and AP1 (23) that control iNOS expression are known *O*-GlcNAc targets. Thus, we hypothesized that there was interaction between *O*-GlcNAc and iNOS that ultimately impacts pro-inflammatory cytokine expression.

**Figure 3:**
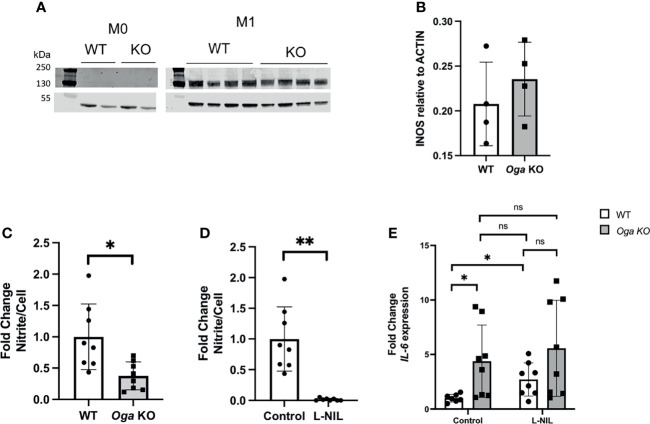
Elevated *O*-GlcNAc inhibits iNOS function. **(A)** Representative western blot to assess iNOS protein levels. **(B)** Quantitation of iNOS protein levels normalized to ACTIN, N = 4. **(C, D)** Media collected after 24 hours of LPS and IFN-γ treatment of BMDMs was used to measure nitrite levels with the Greiss reagent. Levels were normalized to cell number and represented as fold change; **(C)** assessment of nitrite levels in WT (white bar) and *Oga* KO (grey bar); **(D)** assessment of nitrite from WT untreated (control) and L-NIL (iNOS inhibitor) treated WT BMDMs. **(E)** *Il-6* expression was quantified from cells either treated with L-NIL or left untreated (control) at the time of LPS and IFN-γ stimulation from the indicated genotype. Expression was normalized to the geometric mean of *Rplp0* and *Eef2* and represented as fold change. Error bars represent standard deviations. N = 8. *p < 0.05 **p < 0.01, ns, not significant as determined by student’s t-test.

In order to determine if *O-*GlcNAc deregulation impacts iNOS, we first compared protein levels of iNOS in M1 polarized BMDMs derived from *Oga^Vav-Cre^* mice and their wildtype littermates, and did not detect significant changes (**Figures 3A, B**). Knowing that transcriptional expression of iNOS and protein levels were similar between OGA deficient and wildtype cells, we investigated the impact of elevated *O*-GlcNAc on iNOS function through measuring NO production. Using the Greiss reagent, we indirectly measured NO by comparing levels of nitrite in the media collected 24 hours after M1 polarization from wildtype and OGA deficient cells. We found the level of nitrite produced from OGA deficient cells was less than half that as produced from wildtype cells (**Figure 3C**). This finding suggests that elevated *O*-GlcNAc acted to inhibit iNOS function.

In order to test if increased *Il-6* expression in OGA deficient cells was dependent on iNOS-produced NO, we utilized an iNOS specific chemical inhibitor L-NIL. Similar to a previous publication, we found that treating cells with L-NIL inhibited iNOS (**Figure 3D**) and increased expression of *Il-6* in M1 polarized wildtype cells (**Figure 3E**). Further, the increased expression of *Il-6* from wildtype cells treated with L-NIL was similar to expression from M1 polarized OGA deficient cells (**Figure 3E**). To examine if *O*-GlcNAc and iNOS act within the same pathway to regulate *Il-6* expression, we treated OGA deficient cells with L-NIL and found no significant additive effect of the drug (**Figure 3E**). Thus, these data indicated that *O*-GlcNAc acted to impact *Il-6* expression through dampening iNOS function.

### iNOS Can Be *O*-GlcNAc Modified

Protein *O*-GlcNAcylation is known to have a wide range of effects; including control of transcription, translation, protein stability and function (24). iNOS has >50% sequence homology with eNOS and has a conserved threonine, that in eNOS has been defined to be a nutrient-responsive *O*-GlcNAc site (25–27). Thus, knowing that NO production is decreased in *Oga* KO macrophages, we wanted to determine if iNOS itself could be *O*-GlcNAc modified.

**Figure 4:**
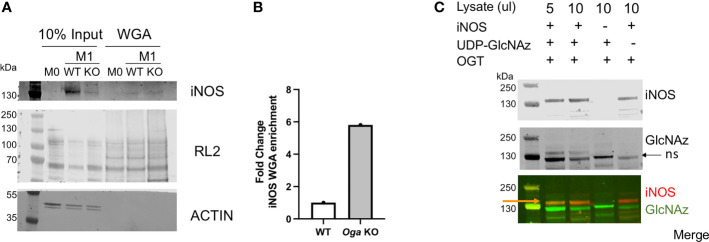
*O*-GlcNAc modification of iNOS. **(A, B)** iNOS and *O*-GlcNAc (RL2) enrichment using wheat germ agglutinin from M0 and M1 polarized WT and *Oga* KO BMDM lysates. Lysates were pooled from cells derived from 3 independent mice of the indicated genotype. **(A)** Blot for iNOS and O-GlcNAc (RL2) in the input and WGA-pull down samples. **(B)** Quantitation of iNOS detected in the WGA enriched lanes normalized to 10% input and represented as fold change. **(C)** *In vitro* OGT assay was performed using bacterial lysates expressing recombinant full-length mouse iNOS (lanes 1,2), bacterial lysates that do not express mouse iNOS (lane 3) or with no nucleotide sugar to indicate background GlcNAz staining (lane 4). After Staudinger ligation, assays were run out on a gel and blotted, with iNOS detected by an iNOS antibody and GlcNAz modification detected with streptavidin conjugate-IRdye800. Orange arrow points to the overlap of iNOS and GlcNAz, black arrow points to non-specific (ns) GlcNAz staining. For full gel image see supplemental figure 3.

First, we took advantage of the ability of wheat germ agglutinin (WGA) to bind to terminal GlcNAc residues and performed WGA pull-down assays using lysates from M0 and M1 polarized wildtype and *Oga* KO BMDMs. After pull-down with WGA and blotting for iNOS we readily detected iNOS bands in the M1 polarized samples, with no band in the M0 sample (**Figures 4A, B**). Further, we detected about a 6 fold enrichment in the amount of iNOS pulled down with WGA when normalized to input for the *Oga* KO cells versus wildtype (**Figures 4A, B**). Thus, iNOS was either directly *O*-GlcNAc modified, or interacted *in vivo* with *O*-GlcNAc modified proteins.

Because the WGA assay did not specifically indicate modification of iNOS itself, we performed an *in vitro* OGT assay using iNOS as the substrate. Here, we incubated recombinant OGT with commercially available bacterial lysates containing full length recombinant mouse iNOS, along with UDP-GlcNAz, similar to the protocol previously published from our laboratory (19). Following a staudinger-ligation, which adds a biotin onto GlcNAzylated proteins, we visualized modified proteins using a streptavidin-conjugated IR dye. Using this *in vitro* assay, we detected direct modification of iNOS (**Figure 4C**). Thus, iNOS either is modified or interacts with modified proteins *in vivo*, and is an OGT substrate *in vitro*.

Taken together, our data indicated that the nutrient responsive *O*-GlcNAc modification acts to support macrophage homeostasis. Through interaction with iNOS, increased *O*-GlcNAc limits NO production and ultimately influences expression of specific inflammatory cytokines like *Il-6*.

## Discussion

Macrophages are characterized by their complex abilities to respond to the local microenvironment. Depending on the stimulus, they can provide pro-inflammatory signals and act as the first line of defense against pathogens, or help maintain cellular and tissue homeostasis with anti-inflammatory “healing” signals. In fact, macrophage polarization is characterized by distinct metabolic profiles indicating that nutrient utilization plays a key role in coordinating proper cellular response. This is underscored by the ability of nutrients like glucose to modulate the inflammatory response of macrophages (10, 11, 17). Further, diseases characterized by deregulated nutrient signaling, like diabetes and other metabolic disorders, often have an inflammatory component. At the nexus of nutrient sensing and cellular signaling is the *O-*GlcNAc modification. Thus, understanding how regulation of *O*-GlcNAcylation impacts the macrophage inflammatory response is of great importance. Previous reports that investigated the impact of *O*-GlcNAc on LPS stimulated BMDMs had not come to clear conclusions as some found enhanced *O*-GlcNAcylation of factors like STAT3 enhanced inflammation (12), while others found that decreased *O*-GlcNAcylation of factors like RIPK and S6K enhanced inflammation (15, 16), indicating that *O*-GlcNAc likely has target specific effects.

While there have been studies that assessed the impact of *Ogt* deletion on the inflammatory response of LPS stimulated mouse derived BMDMs, the consequences of *Oga* deletion had not been fully investigated. In this research report, we derived BMDMs from mice in which *Oga* had been deleted in HSCs. Analysis of these *in vitro* stimulated cells provided evidence that *Oga* deletion enhanced gene expression of specific pro-inflammatory cytokines, including the important factor *Il-6*. IL-6 plays critical roles in the inflammatory reponse through the stimulation of acute phase responses, hematopoiesis and immune reaction. Deregulated continual synthesis of IL-6 has pathological effects on inflammatory and autoimmune diseases (28). In fact, targeting IL-6 has led to important therapeutic approaches for the treatment of rheumatoid arthritis, juvenile idiopathic arthritis and Castleman’s disease (28, 29). Enhanced *Il-6* transcription and secretion was quite surprising as LPS stimulation of *Ogt* knockout BMDMs also resulted in increased IL-6, while the OGA inhibitor thiamet-G caused a decrease in this marker (16). It is important to note that genetic deletion of *Oga* in the mouse is a model of chronic OGA dysfunction, where the cells could potentially adapt and compensate for the loss of OGA, thus thiamet-G treatment and *Oga* deletion have the potential to yield different phenotypes. One way that cells compensate for OGA deficiency is through down regulation of OGT (30, 31). However, we did not detect changes in OGT in our model.

Interestingly, we did not find broad transcriptional upregulation of M1 macrophage markers with elevated *O*-GlcNAc as might be expected if there were changes in NF-κB function. In T-cells the c-Rel subunit of NF-κB has been identified as *O-*GlcNAc modified, with this modification being necessary for transactivation and DNA binding (20). Investigations into the impact of *O*-GlcNAc on RAW264.7 stimulation have also invoked NF-κB function, specifically in regulating transcription of iNOS (13, 17). Although, we did not directly investigate NF-κB function, there were no changes in transcription of known macrophage NF-κB target genes like iNOS, suggesting that NF-κB function was unlikely to be the underlying mechanism of altered cytokine expression in the stimulated OGA deficient BMDMs. Further, we did not detect changes in the metabolic regulator *Acod1*. ACOD1 is induced in M1 activated macrophages to produce itaconate. Itaconate regulates succinate levels and feeds back to limit inflammatory cytokine production (32). ACOD1 can also regulate iNOS through alkylation of GAPDH and limiting aerobic glycolysis (33).

With the production of NO, iNOS is a key component of the inflammatory response in macrophages. NO itself is highly diffusible and has a range of fates; it can break up or inactivate heme-containing enzymes or iron sulfur clusters, it can nitrosylate cysteines, and can combine with superoxide to form ONOO^-^ a potent oxidating and nitrating agent causing DNA damage (2). Although necessary for inflammation, iNOS also acts to auto-regulate and dampen expression of pro-inflammatory cytokines (5). Thus iNOS not only is imperative for killing pathogens, but also contributes to macrophage homeostasis. Due to its homology to eNOS, response to glucose and glucosamine (9, 17), and ability to fine-tune pro-inflammatory cytokine expression, iNOS (5) stood out as a good candidate for further investigation. We examined if the pro-inflammatory phenotype, defined by increased *Il-6* expression, acts through the iNOS pathway and if iNOS itself has the potential to be modified. Using the Griess reagent we detected more than a 50% reduction in nitrite after 24 hours of stimulation from BMDMs derived from *Oga^Vav-Cre^* mice as compared to their wildtype littermates. To directly assess if iNOS function could underly the changes detected in *Il-6* expression, we utilized an iNOS specific inhibitor L-NIL. Like previous studies, we detected an increase in *Il-6* expression with L-NIL treatment of wildtype cells that was similar to levels detected in the OGA deficient cells. Importantly, treating OGA deficient cells with L-NIL did not further impact *Il-6* expression. Knowing that *O*-GlcNAc impacted *Il-6* through iNOS function, we assessed the ability of OGT to modify iNOS. Indeed, iNOS pulled down with WGA using lysates from M1 polarized BMDMs and was modified by OGT *in vitro*. Taken together, these data indicated that elevated *O-*GlcNAcylation acts to dampen iNOS function, further enhancing *Il-6* expression beyond a typical response to pro-inflammatory stimuli.

Further investigation to identify the *O*-GlcNAc modified site on iNOS and how mutation of that site impacts iNOS function will be important to fully untangle the mechanism underlying how *O*-GlcNAc impacts iNOS function. It is also interesting that OGT itself has been suggested to be nitrosylated, with nitrosylation inhibiting function (13). This previous report found that increased levels of NO produced after iNOS induction actually decreased OGT nitrosylation, and thus, increased OGT activity. How nitrosylation of OGT might be affected in the OGA deficient cells and how that might impact function, remains an open question warranting future investigations. Although, iNOS is an important factor impacted by elevated *O-*GlcNAc, it is unlikely the only factor contributing to *Oga* deletion phenotypes, as thousands of proteins are known to be *O*-GlcNAc modified (34).

In this research report we provide evidence that elevated *O*-GlcNAc acts to enhance the inflammatory response of macrophages through increased expression of specific pro-inflammatory cytokines. Further, we found that iNOS is an OGT target, whose function is dampened by increased *O*-GlcNAc. These data underscore the importance of balanced *O*-GlcNAc cycling, as both chronic low levels of *O*-GlcNAc (15, 16) and high levels of *O*-GlcNAc have impacts on the macrophage inflammatory response. Thus, *O*-GlcNAc is a key nutrient sensor whose regulation is integral for immune homeostasis.

## Data Availability Statement

The raw data supporting the conclusions of this article will be made available by the authors, without undue reservation.

## Ethics Statement

The animal study was reviewed and approved by the NIDDK Animal Care and Use Committee, National Institutes of Health.

## Author Contributions

All authors designed experiments and analyzed data. LA performed experiments, wrote the manuscript. All authors read and commented on the manuscript. All authors contributed to the article and approved the submitted version.

## Funding

Funding was provided by NIDDK intramural research program grant ZIADK060103.

## Conflict of Interest

The authors declare that the research was conducted in the absence of any commercial or financial relationships that could be construed as a potential conflict of interest.

## Publisher’s Note

All claims expressed in this article are solely those of the authors and do not necessarily represent those of their affiliated organizations, or those of the publisher, the editors and the reviewers. Any product that may be evaluated in this article, or claim that may be made by its manufacturer, is not guaranteed or endorsed by the publisher.

## Supplementary Material

Supplementary Figure 1 Similar expression of Ogt in M0 and M1 polarized BMDMs. Gene expression of Ogt in M0 and M1 stimulated WT and Oga KO BMDMs. Expression was normalized to the geometric mean of Rplp0 and Eef2 and represented as fold change. N=4-6.

Supplementary Figure 2 No change in gene expression of M2 markers in OGA deficient BMDMs. BMDMs derived from OgaVav-Cre (KO) or wildtype (WT) mouse bone marrow were stimulated with IL-4 (M2 polarization) for 24 hours. Gene expression was assessed for M2 markers with respective genes indicated on the X-axis. Expression was normalized to the geometric mean of Rplp0 and Eef2 and represented as fold change. N=3-4.

Supplementary Figure 3 Full gel from figure 4C. Full gel from figure 4C including lane 5 in which Casein Kinase II (CKII), a well-defined OGT substrate, was used as a positive control for OGT activity.

## References

[1] Murray PJ. Macrophage Polarization. Annu Rev Physiol (2017) 79:541–66. doi: 10.1146/annurev-physiol-022516-034339 27813830

[2] Cinelli MA Do HT Miley GP Silverman RB. Inducible Nitric Oxide Synthase: Regulation, Structure, and Inhibition. Med Res Rev (2020) 40(1):158–89. doi: 10.1002/med.21599 PMC6908786 31192483

[3] Aktan F. iNOS-Mediated Nitric Oxide Production and Its Regulation. Life Sci (2004) 75(6):639–53. doi: 10.1016/j.lfs.2003.10.042 15172174

[4] Hausel P Latado H Courjault-Gautier F Felley-Bosco E. Src-Mediated Phosphorylation Regulates Subcellular Distribution and Activity of Human Inducible Nitric Oxide Synthase. Oncogene (2006) 25(2):198–206. doi: 10.1038/sj.onc.1209030 16116474

[5] Lu G Zhang R Geng S Peng L Jayaraman P Chen C. Myeloid Cell-Derived Inducible Nitric Oxide Synthase Suppresses M1 Macrophage Polarization. Nat Commun (2015) 6:6676. doi: 10.1038/ncomms7676 25813085 PMC4389243

[6] Xiong H Zhu C Li F Hegazi R He K Babyatsky M. Inhibition of Interleukin-12 P40 Transcription and NF-KappaB Activation by Nitric Oxide in Murine Macrophages and Dendritic Cells. J Biol Chem (2004) 279(11):10776–83. doi: 10.1074/jbc.M313416200 14679201

[7] Pavlou S Lindsay J Ingram R Xu H Chen M. Sustained High Glucose Exposure Sensitizes Macrophage Responses to Cytokine Stimuli But Reduces Their Phagocytic Activity. BMC Immunol (2018) 19(1):24. doi: 10.1186/s12865-018-0261-0 29996768 PMC6042333

[8] Jha AK Huang SC Sergushichev A Lampropoulou V Ivanova Y Loginicheva E. Network Integration of Parallel Metabolic and Transcriptional Data Reveals Metabolic Modules That Regulate Macrophage Polarization. Immunity (2015) 42(3):419–30. doi: 10.1016/j.immuni.2015.02.005 25786174

[9] Hwang JS Kwon MY Kim KH Lee Y Lyoo IK Kim JE. Lipopolysaccharide (LPS)-Stimulated iNOS Induction Is Increased by Glucosamine Under Normal Glucose Conditions But Is Inhibited by Glucosamine Under High Glucose Conditions in Macrophage Cells. J Biol Chem (2017) 292(5):1724–36. doi: 10.1074/jbc.M116.737940 PMC5290947 27927986

[10] Jia Y Zheng Z Wang Y Zhou Q Cai W Jia W. SIRT1 Is a Regulator in High Glucose-Induced Inflammatory Response in RAW264. 7 Cells PloS One (2015) 10(3):e0120849. doi: 10.1371/journal.pone.0120849 25793995 PMC4368832

[11] Dissanayake WC Oh JK Sorrenson B Shepherd PR. Glucose Regulates Expression of Pro-Inflammatory Genes, IL-1β and IL-12, Through a Mechanism Involving Hexosamine Biosynthesis Pathway-Dependent Regulation of α-E Catenin. Biosci Rep (2021) 41(7):BSR20211066. doi: 10.1042/BSR20211066 34139004 PMC8243339

[12] Li X Zhang Z Li L Gong W Lazenby AJ Swanson BJ. Myeloid-Derived Cullin 3 Promotes STAT3 Phosphorylation by Inhibiting OGT Expression and Protects Against Intestinal Inflammation. J Exp Med (2017) 214(4):1093–109. doi: 10.1084/jem.20161105 PMC5379975 28280036

[13] Ryu IH Do SI. Denitrosylation of S-Nitrosylated OGT Is Triggered in LPS-Stimulated Innate Immune Response. Biochem Biophys Res Commun (2011) 408(1):52–7. doi: 10.1016/j.bbrc.2011.03.115 21453677

[14] Chang YH Weng CL Lin KI. O-GlcNAcylation and Its Role in the Immune System. J BioMed Sci (2020) 27(1):57. doi: 10.1186/s12929-020-00648-9 32349769 PMC7189445

[15] Li X Gong W Wang H Li T Attri KS Lewis RE. O-GlcNAc Transferase Suppresses Inflammation and Necroptosis by Targeting Receptor-Interacting Serine/Threonine-Protein Kinase 3. Immunity (2019) 50(3):576–90.e6. doi: 10.1016/j.immuni.2019.01.007 30770249 PMC6426684

[16] Yang Y Li X Luan HH Zhang B Zhang K Nam JH. OGT Suppresses S6K1-Mediated Macrophage Inflammation and Metabolic Disturbance. Proc Natl Acad Sci USA (2020) 117(28):16616–25. doi: 10.1073/pnas.1916121117 PMC7368321 32601203

[17] Hwang SY Hwang JS Kim SY Han IO. O-GlcNAc Transferase Inhibits LPS-Mediated Expression of Inducible Nitric Oxide Synthase Through an Increased Interaction With Msin3a in RAW264.7 Cells. Am J Physiol Cell Physiol (2013) 305(6):C601–8. doi: 10.1152/ajpcell.00042.2013 23824843

[18] Abramowitz LK Harly C Das A Bhandoola A Hanover JA. Blocked O-GlcNAc Cycling Disrupts Mouse Hematopoeitic Stem Cell Maintenance and Early T Cell Development. Sci Rep (2019) 9(1):12569. doi: 10.1038/s41598-019-48991-8 31467334 PMC6715813

[19] Kim EJ Abramowitz LK Bond MR Love DC Kang DW Leucke HF. Versatile O-GlcNAc Transferase Assay for High-Throughput Identification of Enzyme Variants, Substrates, and Inhibitors. Bioconjug Chem (2014) 25(6):1025–30. doi: 10.1021/bc5001774 PMC4215860 24866374

[20] Ramakrishnan P Clark PM Mason DE Peters EC Hsieh-Wilson LC Baltimore D. Activation of the Transcriptional Function of the NF-KappaB Protein C-Rel by O-GlcNAc Glycosylation. Sci Signal (2013) 6(290):ra75. doi: 10.1126/scisignal.2004097 23982206 PMC4066889

[21] Zhao M Xiong X Ren K Xu B Cheng M Sahu C. Deficiency in Intestinal Epithelial O-GlcNAcylation Predisposes to Gut Inflammation. EMBO Mol Med (2018) 10(8):e8736. doi: 10.15252/emmm.201708736 29941542 PMC6079539

[22] Jitschin R Böttcher M Saul D Lukassen S Bruns H Loschinski R. Inflammation-Induced Glycolytic Switch Controls Suppressivity of Mesenchymal Stem Cells *via* STAT1 Glycosylation. Leukemia (2019) 33(7):1783–96. doi: 10.1038/s41375-018-0376-6 30679801

[23] Tai HC Khidekel N Ficarro SB Peters EC Hsieh-Wilson LC. Parallel Identification of O-GlcNAc-Modified Proteins From Cell Lysates. J Am Chem Soc (2004) 126(34):10500–1. doi: 10.1021/ja047872b 15327282

[24] Bond MR Hanover JA. O-GlcNAc Cycling: A Link Between Metabolism and Chronic Disease. Annu Rev Nutr (2013) 33:205–29. doi: 10.1146/annurev-nutr-071812-161240 PMC10483992 23642195

[25] He A Hu S Pi Q Guo Y Long Y Luo S. Regulation of O-GlcNAcylation on Endothelial Nitric Oxide Synthase by Glucose Deprivation and Identification of Its O-GlcNAcylation Sites. Sci Rep (2020) 10(1):19364. doi: 10.1038/s41598-020-76340-7 33168911 PMC7652922

[26] Aulak KS Barnes JW Tian L Mellor NE Haque MM Willard B. Specific O-GlcNAc Modification at Ser-615 Modulates eNOS Function. Redox Biol (2020) 36:101625. doi: 10.1016/j.redox.2020.101625 32863226 PMC7334407

[27] Li C He A Guo Y Yang X Luo M Cheng Z. Hypertonic Stress Modulates eNOS Function Through O-GlcNAc Modification at Thr-866. Sci Rep (2021) 11(1):11272. doi: 10.1038/s41598-021-90321-4 34050207 PMC8163736

[28] Tanaka T Narazaki M Kishimoto T. IL-6 in Inflammation, Immunity, and Disease. Cold Spring Harb Perspect Biol (2014) 6(10):a016295. doi: 10.1101/cshperspect.a016295 25190079 PMC4176007

[29] Choy EH De Benedetti F Takeuchi T Hashizume M John MR Kishimoto T. Translating IL-6 Biology Into Effective Treatments. Nat Rev Rheumatol (2020) 16(6):335–45. doi: 10.1038/s41584-020-0419-z PMC7178926 32327746

[30] Pravata V Omelková M Stavridis M Desbiens C Stephen H Lefeber D. An Intellectual Disability Syndrome With Single-Nucleotide Variants in O-GlcNAc Transferase. Eur J Hum Genet (2020) 28(6):706–14. doi: 10.1038/s41431-020-0589-9 PMC7253464 32080367

[31] Park S Zhou X Pendleton K Hunter O Kohler J O'Donnell K. A Conserved Splicing Silencer Dynamically Regulates O-GlcNAc Transferase Intron Retention and O-GlcNAc Homeostasis. Cell Rep (2017) 20(5):1088–99. doi: 10.1016/j.celrep.2017.07.017 PMC5588854 28768194

[32] Lampropoulou V Sergushichev A Bambouskova M Nair S Vincent EE Loginicheva E. Itaconate Links Inhibition of Succinate Dehydrogenase With Macrophage Metabolic Remodeling and Regulation of Inflammation. Cell Metab (2016) 24(1):158–66. doi: 10.1016/j.cmet.2016.06.004 PMC5108454 27374498

[33] Wu R Chen F Wang N Tang D Kang R. ACOD1 in Immunometabolism and Disease. Cell Mol Immunol (2020) 17(8):822–33. doi: 10.1038/s41423-020-0489-5 PMC7395145 32601305

[34] Ma J Hart GW. O-GlcNAc Profiling: From Proteins to Proteomes. Clin Proteomics (2014) 11(1):8. doi: 10.1186/1559-0275-11-8 24593906 PMC4015695

