# Supplementary figures and images for: Chronically Elevated O-GlcNAcylation Limits Nitric Oxide Production and Deregulates Specific Pro-Inflammatory Cytokines
Source: Front Immunol. 2022 Apr 1;13:802336. doi: 10.3389/fimmu.2022.802336 (PMC9010940; doi:10.3389/fimmu.2022.802336)

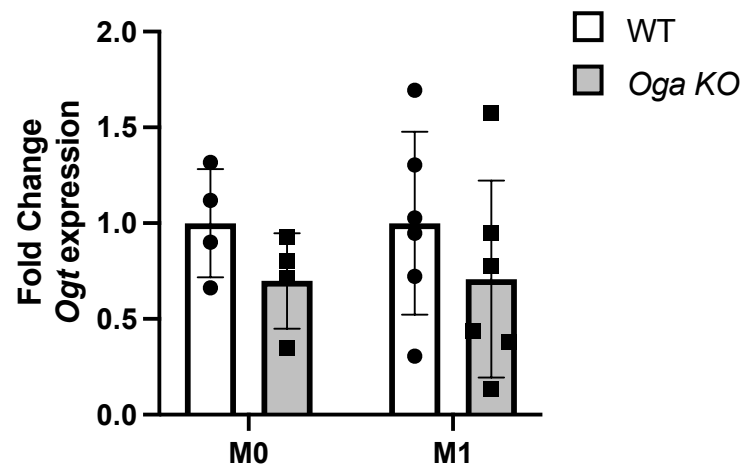

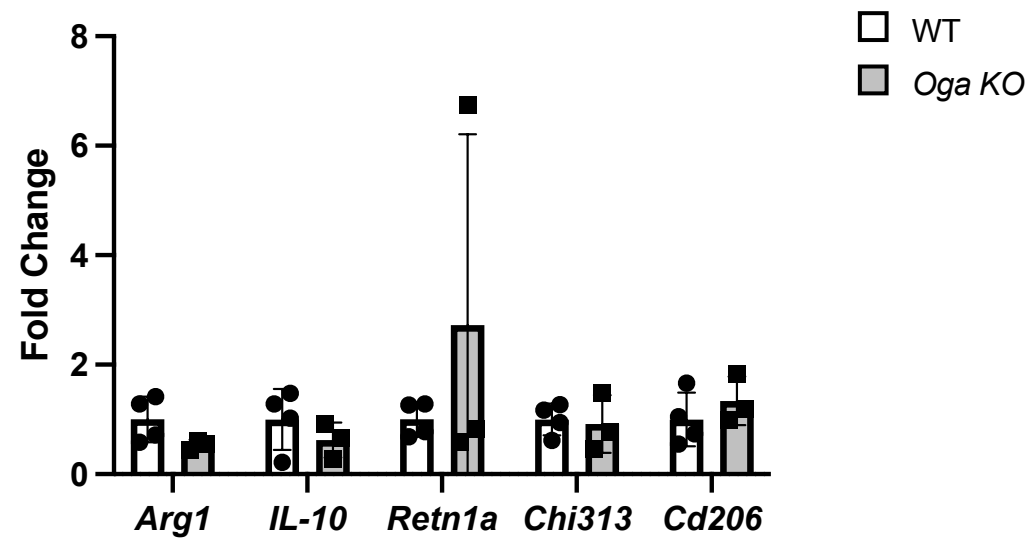

|             |   |    |    |    |   |
|-------------|---|----|----|----|---|
| Lysate (ul) | 5 | 10 | 10 | 10 | - |
| iNOS        | + | +  | -  | +  | - |
| UDP-GlcNAz  | + | +  | +  | -  | + |
| OGT         | + | +  | +  | +  | + |
| CKII        | - | -  | -  | -  | + |

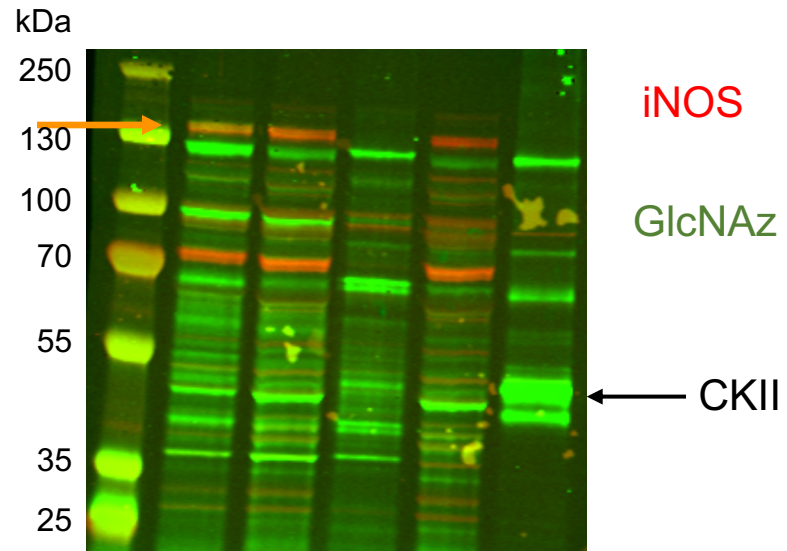

Supplement: Supplementary Figure 1 — Similar expression of Ogt in M0 and M1 polarized BMDMs. Gene expression of Ogt in M0 and M1 stimulated WT and Oga KO BMDMs. Expression was normalized to the geometric mean of Rplp0 and Eef2 and represented as fold change. N=4-6. [file Image_1.pdf]
