# Supplementary material for: Chronically Elevated O-GlcNAcylation Limits Nitric Oxide Production and Deregulates Specific Pro-Inflammatory Cytokines
Source: Front Immunol. 2022 Apr 1;13:802336. doi: 10.3389/fimmu.2022.802336 (PMC9010940; doi:10.3389/fimmu.2022.802336)
Supplement: Supplementary file 2 [file Table_1.pdf]

Genes	Sense (5'-3')	Antisense (5'-3')
Oga	GTGCAGTGGTTAGGGTGTCG	AGCAAACGCTGGAACTCTCC
Ogt	CAGAGAGGCATGGGAATCTC	TGCATGCCTGGAATAGACTG
Il6	TAGTCCTTCCTACCCCAATTTCC	TTGGTCCTTAGCCACTCCTTC
Tnfα	GTCAGGTTGCCTCTGTCTCA	TCAGGGAAGAGTCTGGAAAG
Il1β	CTTCAGGCAGGCAGTATCAC	CCAGCAGGTTATCATCATCATCC
Il12	GGAAGCACGGCAGCAGAATAA	CTTGAGGGAGAAGTAGGAATG
Ifnβ	CGTGGGAGATGTCCTCAACT	AGATCTCTGCTCGGACCACC
Cxcl9	GGAACCCTAGTGATAAGGAATGCA	TGAGGTCTTTGAGGGATTTGTAGTG
Cxcl10	GACGGTCCGCTGCAACTG	CTTCCCTATGGCCCTCATTCT
Ccl2	CCTGCTGCTACTCATTCACC	ATGTCTGGACCCATTCCTTC
Ccl5	CCTGCTGCTTTGCCTACCTC	GAATCAAGAAACCCTCTATCCTA
Cd86	TTGTGTGTGTTCTGGAAACGGAG	AACTTAGAGGCTGTGTTGCTGGG
Nos2	CCCTTCAATGGTTGGTACATGG	ACATTGATCTCCGTGACAGCC
Irf5	GGTCAACGGGGAAAAGAAACT	CATCCACCCCTTCAGTGTACT
Irf3	GGCTTGTGATGGTCAAGGTT	CATGTCCTCCACCAAGTCCT
Stat3	AATGGAAATTGCCCGGATC	AGGCGAGACTCTTCCCACAG
Socs1	CCTCCTCGTCCTCGTCTTC	GAAAAGGCAGTCGAAGGTCTC
Socs3	ACCTTCAGCTCCAAAAGCGAGTAC	CGCTCCAGTAGAATCCGCTCTC
Acod1	TTTGGGGTCGACCAGACTTC	CCATGGAGTGAACAGCAACAC
Rplp0	TCCCACTTACTGAAAAGGTCAAG	TCCGACTCTTCCTTTGCTTC
Eef2	AGGCCGCCATGGGTATTAAG	AAGGCATAGAAGCGGCCTTT
Arg1	GAACACGGCAGTGGCTTTAAC	TGCTTAGCTCTGTCTGCTTTG
Il10	GCTCTTACTGACTGGCATGAG	CGCAGCTCTAGGAGCATGTG
Retn1a	CCAATCCAGCTAACTATCCCTCC	ACCCAGTAGCAGTCATCCCA
Chi313	GTCTTGCTCATGTGTGTAAGTGA	CAGGTCTGGCAATTCTTCTGAA
Cd206	TCTTTGCCTTTCCCAGTCTCC	TGACACCCAGCGGAATTTC

Supplemental Table 1: Primer sequences for qRT-PCR
